# Whole-genome CRISPR screening identifies PI3K/AKT as a downstream component of the oncogenic GNAQ–focal adhesion kinase signaling circuitry

**DOI:** 10.1016/j.jbc.2022.102866

**Published:** 2022-12-31

**Authors:** Nadia Arang, Simone Lubrano, Damiano Cosimo Rigiracciolo, Daniela Nachmanson, Scott M. Lippman, Prashant Mali, Olivier Harismendy, J. Silvio Gutkind

**Affiliations:** 1 University of California San Diego, Moores Cancer Center, La Jolla, California, USA; 2 University of California San Diego, Biomedical Sciences Graduate Program, La Jolla, California, USA; 3 Department of Pharmacology, University of California San Diego, School of Medicine, La Jolla, California, USA; 4 Department of Pharmacy, University of Pisa, Pisa, Italy; 5 University of California San Diego, Bioinformatics and Systems Biology Graduate Program, La Jolla, California, USA; 6 Department of Bioengineering, University of California San Diego, La Jolla, California, USA; 7 Division of Biomedical Informatics, Department of Medicine, University of California San Diego, La Jolla, California, USA

**Keywords:** G protein, GNAQ/GNA11, FAK, Signal transduction, Protein phosphorylation, Uveal melanoma, CNO, clozapine-*N*-oxide, EGF, epidermal growth factor, ERK, extracellular signal–regulated kinase, FAK, focal adhesion kinase, FAKi, FAK inhibitor, GPCR, G protein–coupled receptor, HEK293, human embryonic kidney 293 cell line, IP, immunoprecipitation, MAPK, mitogen-activated protein kinase, mTOR, mammalian target of rapamycin, mUM, metastatic UM, pERK, phosphorylated ERK, pFAK, phosphorylated FAK, PI3K, phosphoinsositide-3-kinase, PTEN, phosphatase and tensin homolog, sgRNA, single-guide RNA, TSC2, tuberous sclerosis complex 2, UM, uveal melanoma, YAP, Yes-associated protein

## Abstract

G proteins and G protein–coupled receptors activate a diverse array of signal transduction pathways that promote cell growth and survival. Indeed, hot spot–activating mutations in GNAQ/GNA11, encoding Gαq proteins, are known to be driver oncogenes in uveal melanoma (UM), for which there are limited effective therapies currently available. Focal adhesion kinase (FAK) has been recently shown to be a central mediator of Gαq-driven signaling in UM, and as a result, is being explored clinically as a therapeutic target for UM, both alone and in combination therapies. Despite this, the repertoire of Gαq/FAK-regulated signaling mechanisms have not been fully elucidated. Here, we used a whole-genome CRISPR screen in GNAQ-mutant UM cells to identify mechanisms that, when overactivated, lead to reduced sensitivity to FAK inhibition. In this way, we found that the PI3K/AKT signaling pathway represented a major resistance driver. Our dissection of the underlying mechanisms revealed that Gαq promotes PI3K/AKT activation *via* a conserved signaling circuitry mediated by FAK. Further analysis demonstrated that FAK activates PI3K through the association and tyrosine phosphorylation of the p85 regulatory subunit of PI3K and that UM cells require PI3K/AKT signaling for survival. These findings establish a novel link between Gαq-driven signaling and the stimulation of PI3K as well as demonstrate aberrant activation of signaling networks underlying the growth and survival of UM and other Gαq-driven malignancies.

G protein–coupled receptors (GPCRs) and their associated G proteins are the largest family of cell surface proteins involved in signal transduction. As a result, they are central mediators of numerous cellular and physiological processes (1, 2). Most GPCRs activate one or multiple Gα protein families: Gαi, Gα12, Gαs, and Gαq, each activating distinct signaling pathways (3). Remarkably, recent analyses have revealed that G proteins and GPCRs are mutated in nearly 30% of all human cancers (4, 5). In particular, hot spot mutations in *GNAQ* and *GNA11*, referred to as *GNAQ* oncogenes, encoding GTPase-deficient and constitutively active Gαq proteins, have been identified in ∼93% of uveal melanoma (UM) and 4% of skin cutaneous melanoma, where they act as driver oncogenes (6, 7, 8, 9, 10).

UM is the most common primary cancer of the eye in adults and is the second most common melanoma subtype after skin cutaneous melanoma (11). Approximately 50% of UM patients develop metastatic UM (mUM), most of which are refractory to current therapies, leading to patient death within a year (12). The mitogen-activated protein kinase (MAPK)/extracellular signal–regulated kinase (ERK) inhibitors selumetinib and trametinib have been extensively evaluated for mUM treatment; however, MAPK/extracellular signal–regulated kinase inhibition with these agents has nearly no impact on the overall survival of mUM patients (13, 14, 15). Recent studies exploring the use of tebentafusp, a bispecific fusion antibody, have shown significant yet limited increases in patient overall survival, leading to Food and Drug Administration approval in unresectable or mUM patients (16, 17). Despite this, there is still an urgent need for novel and effective therapeutic strategies for advanced UM and mUM. This prompted renewed interest in investigating the mechanisms by which prolonged Gαq signaling controls cancer cell growth, toward identifying novel pharmacological targets for therapeutic intervention in UM.

The precise molecular mechanisms by which oncogenic Gαq transduce sustained proliferative signals is not yet fully defined. This is primarily because of the large number of second messenger–generating systems and signaling events perturbed upon Gαq activation (18, 19). Recent findings support that mutant Gαq activates phospholipase C β/PKC, leading to the activation of ERK/MAPK, while concomitantly stimulating an exchange factor TRIO, thereby activating a Rho GTPase signaling circuitry (8, 20, 21). The latter activates Yes-associated protein (YAP), a transcriptional coactivator regulated by the Hippo pathway (9). Of interest, synthetic lethal gene interactions of Gαq revealed that downstream of the Gαq-TRIO–RhoA–ROCK pathway, focal adhesion kinase (FAK), a nonreceptor tyrosine kinase, is a central mediator of noncanonical Gαq-driven signaling and a druggable signaling node downstream of the *GNAQ* oncogene (22).

Although the precise mechanisms leading to activation of FAK by Gαq have yet to be determined, these studies provided a direct link between Gαq-FAK initiated tyrosine phosphorylation networks and YAP activation, driving UM growth. As targeting FAK in UM is now being advanced to the clinic, we hypothesize that elucidation of the Gαq-FAK-regulated signaling networks may help identify novel downstream targets of Gαq, some of which may represent mechanisms that should be targeted to optimize therapeutic responses to FAK inhibitor (FAKi). Toward this end, we aimed at investigating additional Gαq-FAK-regulated signaling circuitries that may be critical to promote growth in UM and other Gαq-driven malignancies.

## Results

**Figure 1:**
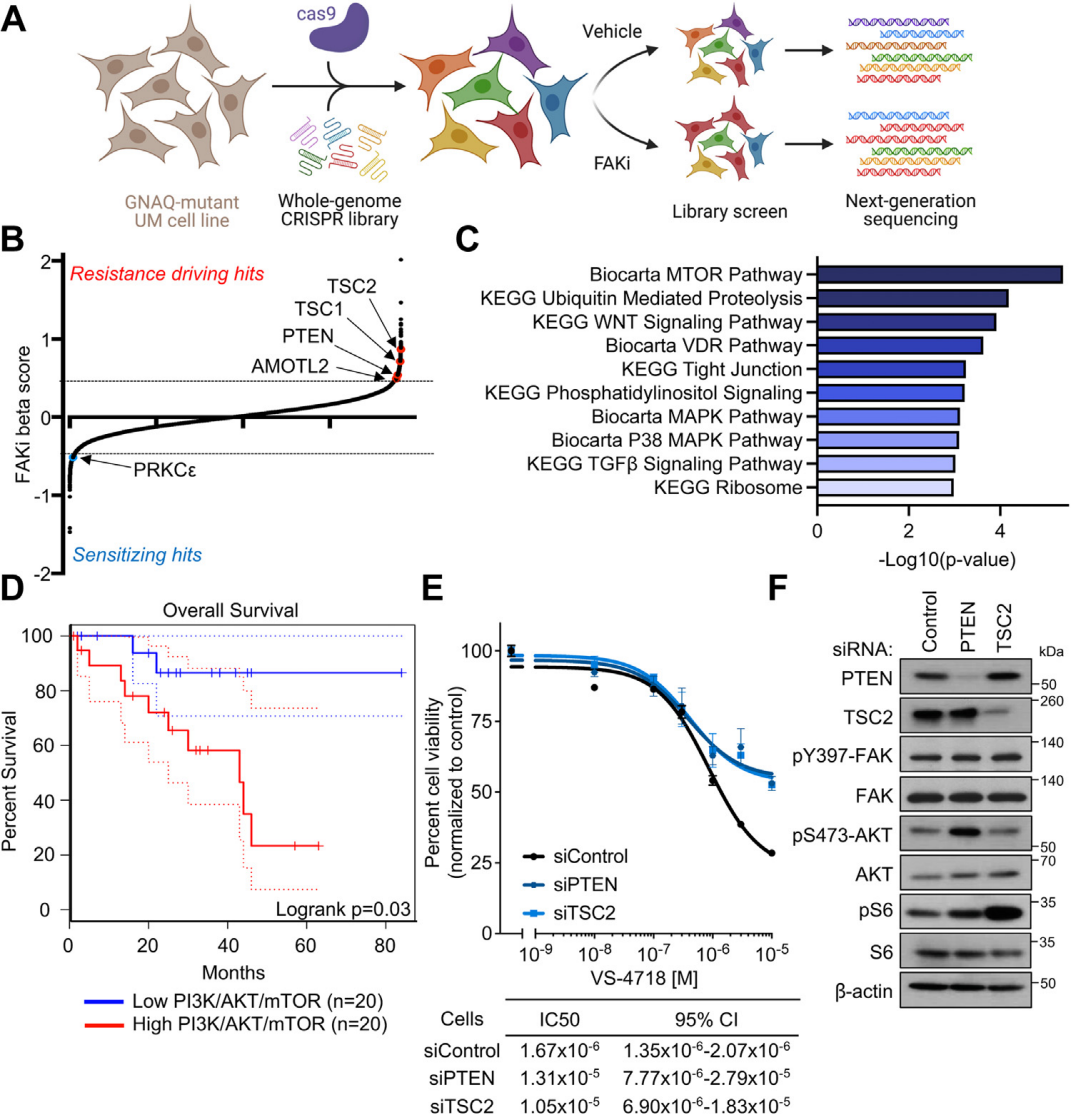
**PI3K/AKT pathway activation drives resistance to FAKi in GNAQ-mutant UM.***A*, schematic of whole-genome CRISPR screen experimental design. Created with Biorender.com. *B*, cell viability represented by beta score where a positive beta score indicates positive selection (resistance) (beta >0.5, indicated by *dotted line*), and a negative beta score indicates negative selection (sensitivity) (beta <−0.5, indicated by *dotted line*) under FAKi treatment. *C*, over-representation analysis of top sgRNAs (FDR <0.015) with positive beta score using KEGG and Biocarta gene sets. Color intensity of bars fade by decreasing −Log_10_*p* value. *D*, overall survival analysis of UM The Cancer Genome Atlas patient cohort with high (*top* 25%) or low (*bottom* 25%) expression of PI3K/AKT/mTOR Hallmark gene signature. *Dotted lines* indicate 95% confidence interval. *E*, cell viability of 92.1 UM cells after siRNA knockdown of PTEN or TSC2 compared with control siRNA in response to VS-4718 (FAKi) treatment for 72 h, percent viability is normalized to vehicle treatment (mean ± SD, n = 3). *F*, phosphorylation of FAK, AKT, and S6 after siRNA-mediated knockdown of CRISPR top hits (PTEN and TSC2) in 92.1 UM cells. Representative immunoblots are shown from n = 3 independent experiments. FAKi, focal adhesion kinase inhibitor; FDR, false discovery rate; KEGG, Kyoto Encyclopedia of Genes and Genomes; mTOR, mammalian target of rapamycin; PTEN, phosphatase and tensin homolog; sgRNA, single-gide RNA; TSC2, tuberous sclerosis complex 2; UM, uveal melanoma.

In order to profile the genetic interactome of Gαq-FAK signaling in UM, we performed a genome-wide CRISPR kKO screen in *GNAQ*-mutant UM cells in the context of FAK inhibition (Fig. 1*A*). Using Cas9-expressing 92.1 UM cells (92.1^Cas9^), infected with the Brunello Human Genome pooled single-guide RNA (sgRNA) library, cells were passaged under 0.1 μM VS-4718 (FAKi) treatment, or vehicle for a total of 19 cell doublings. In order to evaluate pathways that when modulated, resulted in resistance to inhibition of FAK, we examined sgRNAs enriched in FAKi treatment condition. Among the top hits were phosphatase and tensin homolog (PTEN) and tuberous sclerosis complex 2 (TSC2), which are canonical negative regulators of the PI3K/AKT pathway (23), suggesting that enhanced PI3K/AKT signaling could drive resistance to FAKi (Fig. 1*B*). Aligned with this, genes involved in the PI3K/AKT/mammalian target of rapamycin (mTOR) signaling pathway were enriched targets of the sgRNAs yielding the most resistance (Fig. 1*C*, S1*A*). We also observed enrichment of cells with AMOTL2 sgRNAs in the FAKi conditions, which is a negative regulator of the Hippo/YAP pathway, and is aligned with the role of YAP as a downstream target of FAK in UM (22). Conversely, we observed depletion of sgRNAs for PRKCE, which we have demonstrated to be synthetic lethal with FAK (24). Interestingly, increased expression of PI3K/AKT/mTOR gene signature was associated with poor overall survival in The Cancer Genome Atlas UM patients (log-rank test; *p* = 0.03) (Fig. 1*D*). To validate the findings of our CRISPR screen, we performed siRNA-mediated knockdown of the top two PI3K/AKT pathway hits from our screen, PTEN and TSC2, and evaluated the effect on cell viability in response to FAK inhibition (Fig. 1*E*). We found that knockdown of PTEN and TSC2 both resulted in decreased sensitivity to FAKi in UM cells. We next evaluated the effect on PI3K and FAK signaling caused by PTEN and TSC2 loss (Fig. 1*F*, S1*B*). In both cases, while siRNA-mediated knockdown of PTEN and TSC2 resulted in increase in phosphorylation of downstream pathway members, AKT and S6 respectively, the latter often used to monitor mTOR activity (23), it did not lead to a change in phosphorylation of FAK. This suggests that increased PI3K/AKT signaling does not confer resistance to FAKi through FAK reactivation and instead raises the possibility that PI3K/AKT may represent a critical signaling pathway activated by Gαq through FAK.

**Figure 2:**
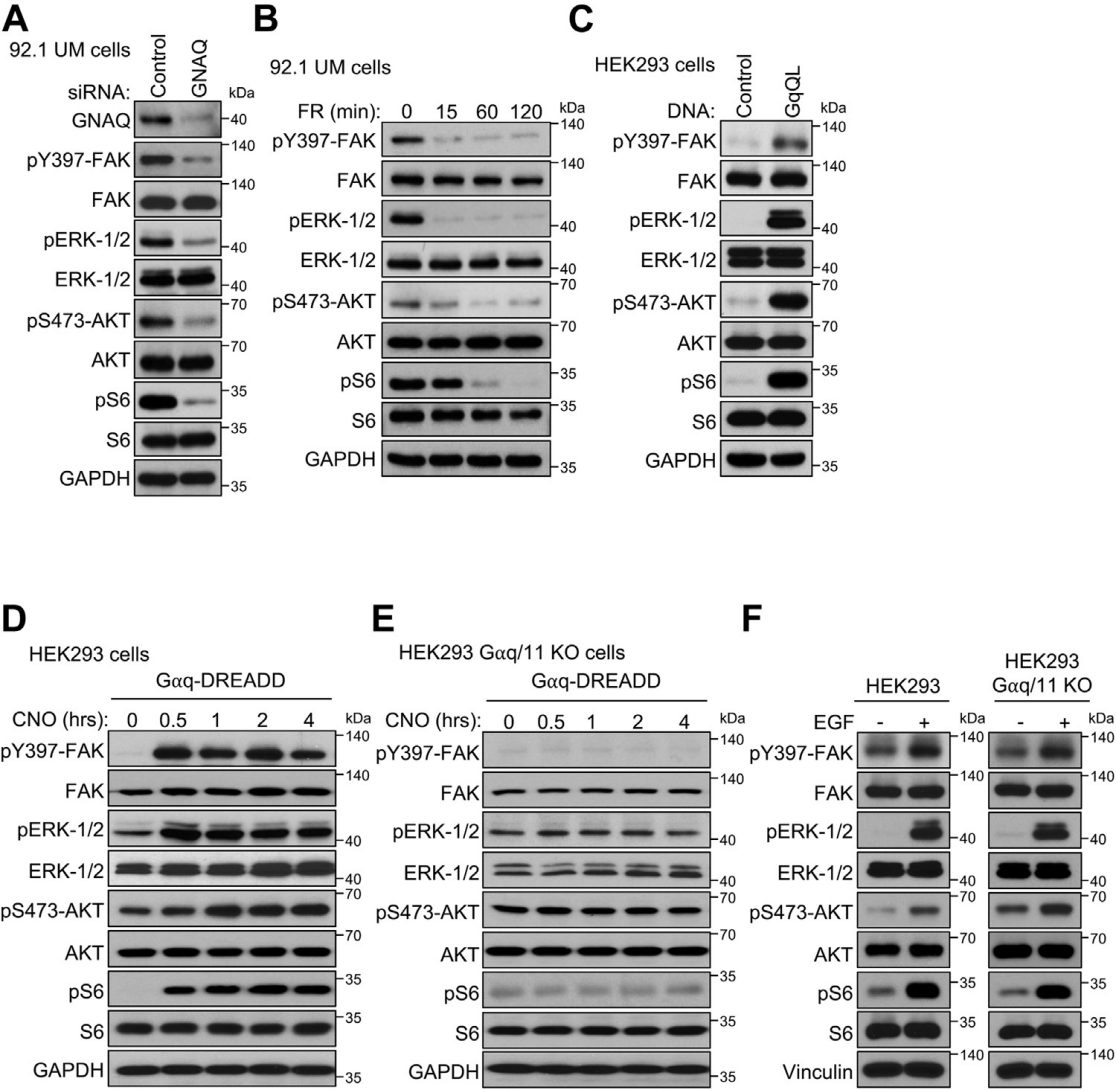
**GNAQ is a regulator of PI3K/AKT signaling.** Phosphorylation of canonical (ERK) and noncanonical (FAK) Gαq-regulated pathways and PI3K/AKT pathway (AKT and S6) in response to *A*, siRNA-mediated knockdown of GNAQ in 92.1 UM cells. *B*, 500 nM FR900359 (Gαq inhibitor) treatment over a time course. *C*, expression of GαqQL in HEK293 cells. *D*, stimulation of Gαq signaling using 1 μM CNO over a time course, after expression of Gαq-DREADD in HEK293 cells or *E*, in Gαq/11 KO HEK293 cells. *F*, phosphorylation of canonical (ERK) and noncanonical (FAK) Gαq-regulated pathways and PI3K/AKT pathway (AKT and S6) in response to 20 nM EGF treatment for 1 h in HEK293 and HEK293 Gαq/11 KO cells. In all cases, representative immunoblots are shown from n = 3 independent experiments. CNO, clozapine-*N*-oxide; EGF, epidermal growth factor; ERK, extracellular signal–regulated kinase; FAK, focal adhesion kinase; HEK293, human embryonic kidney 293 cell line; UM, uveal melanoma.

In this case, however, whether Gαq activates or inhibits PI3K/AKT is not clear, and the overall underlying mechanisms involved are poorly understood (25, 26, 27, 28). Based on these findings, we asked whether the PI3K pathway acts downstream of Gαq-FAK, or if it represents a parallel signaling axis. Inhibiting Gαq with siRNA-mediated knockdown and by pharmacological inhibition with FR900359, resulted in sustained inhibition of canonical (ERK) and noncanonical (FAK) Gαq-driven signaling, as previously reported (24), concomitant with a decreased phosphorylation of the PI3K signaling targets, AKT and S6 (Fig. 2, *A* and *B*, Fig S2, *A*–*D*). However, we did not observe a decrease in the same signaling targets upon pharmacological inhibition of Gαq in a non-Gαq-dependent cutaneous melanoma cell line, SKMEL-28 (Fig. S2*E*). This suggests that Gαq controls PI3K signaling in UM cells harboring active Gαq. As an orthogonal approach, we found that GαqQL, the active Gαq mutant found in UM, promoted the accumulation of the phosphorylated forms of ERK (pERK), FAK (pFAK), AKT, and S6 in human embryonic kidney 293 (HEK293) cells, demonstrating the direct ability of Gαq to promote PI3K/AKT signaling (Fig. 2*C*, S2*F*). We also challenged our observations using the expression of a synthetic Gαq-coupled receptor, termed Gαq-DREADD, which can only be activated by addition of a pharmacologically inert ligand, clozapine-*N*-oxide (CNO) (29, 30). Expression of Gαq-DREADD in HEK293 cells and stimulation with CNO revealed a rapid and sustained increase in pERK and pFAK, in addition to an increase in pAKT and pS6 (Fig. 2*D*). We validated the specificity of this approach by expressing Gαq-DREADD in Gαq/11 KO cells and stimulating with CNO; however, we did not observe an increase in the phosphorylation state of any of the proteins tested (Fig. 2*E*). Challenging both HEK293 and HEK293 Gαq/11 KO cell lines with epidermal growth factor (EGF) treatment revealed an increase in phosphorylation of all tested proteins in both cases, demonstrating the signaling competence in both models (Fig. 2*F*). Collectively, these results indicated that Gαq promotes PI3K/AKT signaling when activated by GPCRs or as part of constitutive Gαq signaling, such as in UM.

**Figure 3:**
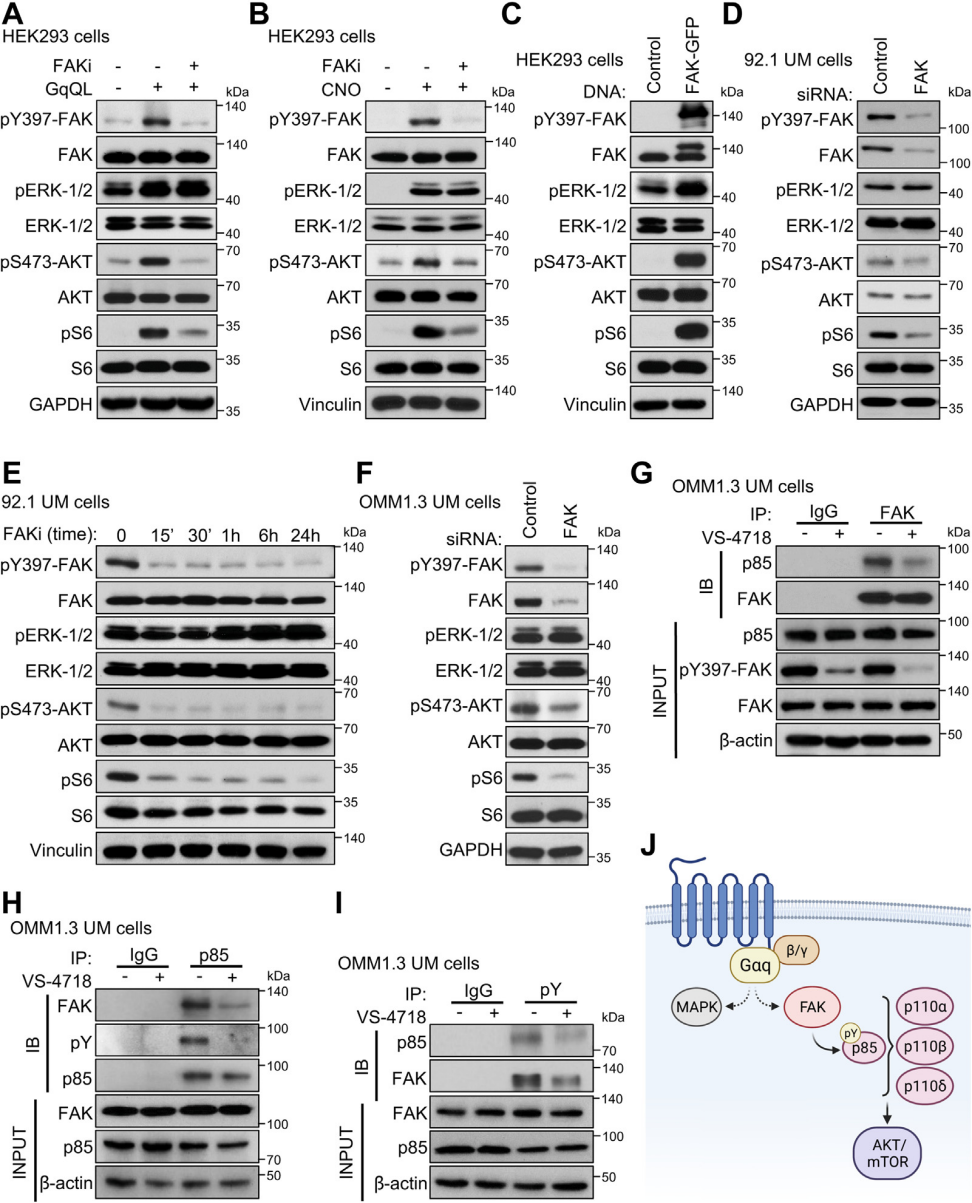
**FAK mediates PI3K/AKT pathway activation through p85 phosphorylation.** Phosphorylation of canonical (ERK) and noncanonical (FAK) Gαq-regulated pathways and PI3K/AKT pathway (AKT and S6) in response to *A*, expression of GαqQL alone or in combination with 1 μM VS-4718 treatment for 15 h in HEK293 cells. *B*, stimulation of Gαq signaling using 1 μM M CNO for 1 h after expression of Gαq-DREADD, in combination with 2 μM VS-4718. *C*, expression of FAK-GFP in HEK293 cells. *D*, siRNA-mediated knockdown of FAK in 92.1 UM cells. *E*, time course of 1 μM VS-4817 treatment in 92.1 UM cells. *F*, siRNA-mediated knockdown of FAK in OMM1.3 UM cells. *G*, association of p85 with FAK after FAK immunoprecipitation with or without 1 μM VS-4718 treatment for 15 h in OMM1.3 UM cells. *H*, association of p85 with FAK and tyrosine phosphorylation after p85 immunoprecipitation and treatment with or without 1 μM VS-4718 treatment for 15 h in OMM1.3 UM cells. *I*, association of p85 and FAK after pY immunoprecipitation with or without 1 μM VS-4817 treatment for 15 h in OMM1.3 UM cells. *J*, schematic of signaling mechanisms regulated by Gαq- and FAK-mediated control of PI3K. Created with Biorender.com. In all cases, representative immunoblots are shown from n = 3 independent experiments. CNO, clozapine-*N*-oxide; ERK, extracellular signal–regulated kinase; FAK, focal adhesion kinase; HEK293, human embryonic kidney 293 cell line.

Based on these findings linking Gαq to enhanced PI3K/AKT activity, we then asked whether Gαq controls PI3K/AKT signaling *via* FAK. To test this, we expressed GαqQL in HEK293 cells alone or in combination with pharmacological inhibition of FAK (Fig. 3*A*). Indeed, inhibition of FAK in the context of Gαq activation was sufficient to block an increase in pAKT and pS6, whereas no change in pERK was observed. Likewise, activation of Gαq using Gαq-DREADD and stimulation with CNO, in combination with FAK inhibition, abrogated an increase in pAKT and pS6 (Fig. 3*B*). Based on these findings, we tested the ability of FAK expression to drive PI3K/AKT signaling. Overexpression of FAK in HEK293 cells led to a potent increase in pAKT and pS6 (Fig. 3*C*, S3*A*). Conversely, blockade of FAK in UM cells with high basal Gαq-FAK activity, using siRNA-mediated knockdown, or by a pharmacological inhibition led to a decrease in pAKT and pS6 levels (Fig. 3, *D*–*F*, Fig. S3, *B*–*D*). These data suggest that in UM cells, persistent Gαq-driven signaling promotes PI3K pathway signaling *via* FAK.

The p110 catalytic subunit of the PI3K heterodimer is comprised of four different isoforms: PI3Kα, PI3Kβ, PI3Kγ, and PI3Kδ. Class IA PI3Ks (α, β, and δ) consist of heterodimers of a catalytic p110 subunit and regulatory p85 subunit (31). In response to stimuli, inhibition of p110 by p85 can be relieved by direct tyrosine phosphorylation of p85 or by recruitment of p85 to tyrosine-phosphorylated motifs on other proteins (31). This prompted us to ask if FAK could associate with and tyrosine phosphorylate p85 directly. By performing coimmunoprecipitation (co-IP) of FAK and p85 in UM cells, we observed strong binding of FAK to p85 under basal conditions that was diminished with FAKi treatment (Fig. 3*G*). The reverse could also be observed, where under basal conditions, co-IP of p85 revealed strong association with FAK that was relieved upon FAKi treatment (Fig. 3*H*). We also observed strong basal tyrosine phosphorylation of p85 that was diminished concomitant with a dissociation from FAK by FAKi treatment. We validated our findings by global IP of tyrosine-phosphorylated proteins using pTyr antibodies in UM cells (Fig. 3*I*). Aligned with our previous results, IP of total pTyr resulted in pulldown of p85 and FAK. Inhibition of FAK activity with FAKi similarly reduced the levels of tyrosine pFAK and p85 available to be extracted by pTyr. Taken together, these findings suggest that Gαq signaling promotes PI3K/AKT pathway activity through FAK-dependent tyrosine phosphorylation and association with PI3K-p85 (Fig. 3*J*).

**Figure 4:**
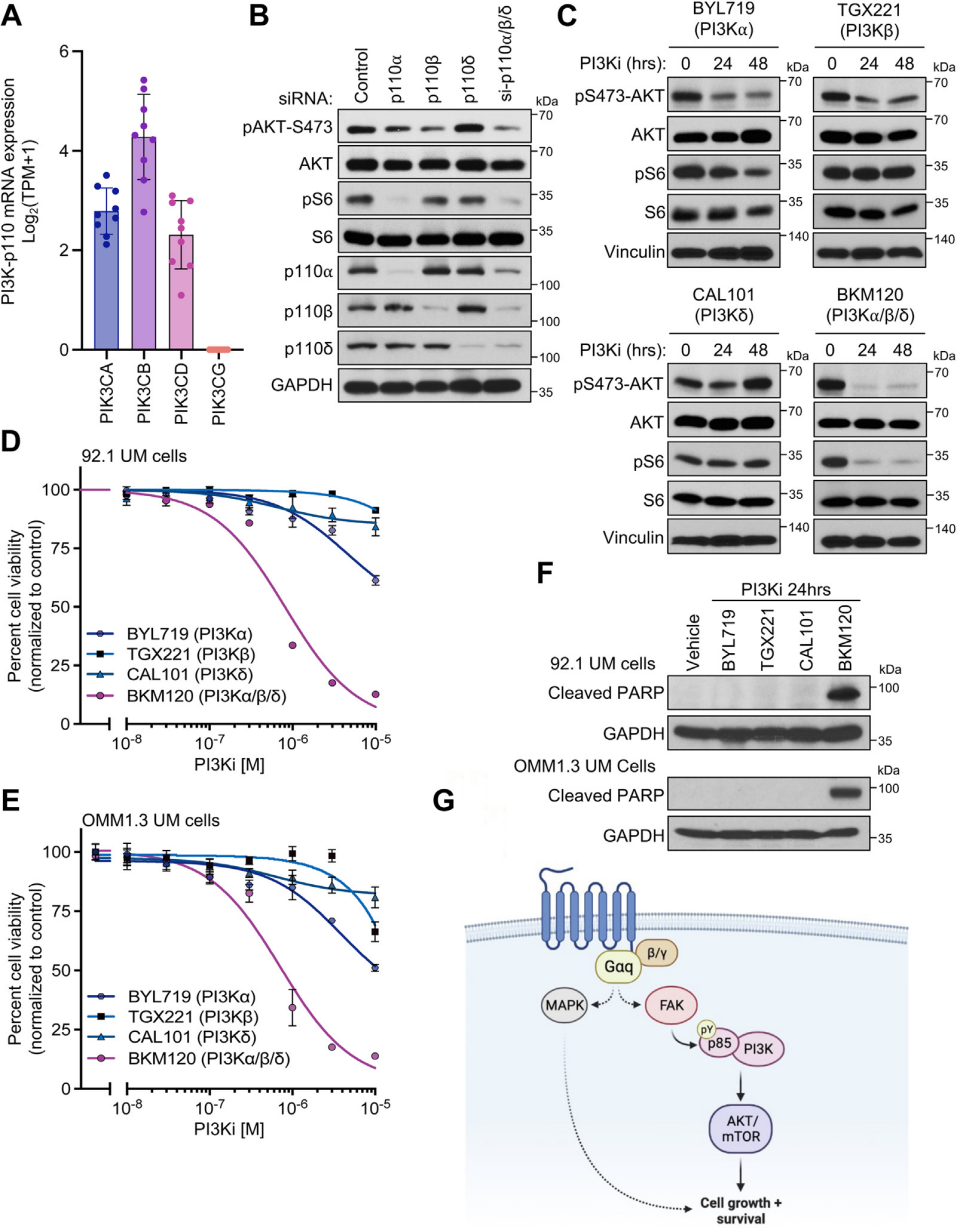
**UM cells are dependent on PI3K/AKT signaling for growth and survival.***A*, mRNA expression of PI3K-p110 isoforms from UM cell lines in DepMap portal, Expression Public 2Q22 (mean ± SD, n = 9 cell lines). *B*, phosphorylation of AKT and S6 after single and combination of siRNA-mediated PI3K-p110 knockdown in OMM1.3 UM cells. *C*, phosphorylation of AKT and S6 after treatment with 1 μM BYL719, TGX221, CAL101, and BKM120 for the indicated time points. *D*, cell viability of 92.1 UM cells and *E*, OMM1.3 UM cells after 72 h treatment with BYL719, TGX221, CAL101, and BKM120. Percent viability is normalized to vehicle treatment (mean ± SD, n = 3). *F*, immunoblot showing cleaved PARP levels in response to treatment with 1 μM PI3Ki as indicated or vehicle control for 24 h in 92.1 (*top*) or OMM1.3 (*bottom*) UM cells. Representative immunoblots are shown from n = 3 independent experiments. *G*, schematic of signaling mechanisms controlled by Gαq. Created with Biorender.com. In all cases, representative immunoblots are shown from n = 3 independent experiments. PARP, uveal melanoma; UM, uveal melanoma.

While expression patterns of each PI3K catalytic subunit isoforms varies across tissues, the expression and isoform usage of PI3K is not currently known in UM. We first screened expression of each PI3K-p110 isoform in a number of UM cell lines on the DepMap portal and found that with the exception of PI3Kγ, all p110 isoforms were expressed (Fig. 4*A*). We next performed siRNA-mediated knockdown of the major UM-associated p110 isoforms alone, and in combination, and assessed levels of PI3K pathway activity by measuring pAKT and pS6 (Fig. 4*B*). We found that in UM cells, PI3Kα and PI3Kβ were major drivers of PI3K signaling, with the strongest reduction in the context of triple p110 isoform knockdown. To complement our genetic knockdown approach, we tested the ability of p110 isoform-specific as well as a pan-PI3K pharmacological inhibitor to inhibit AKT/S6. Aligned with our knockdown data, only BKM120, the pan-PI3K inhibitor that we tested, was able to reduce both pAKT and pS6 in a potent and sustained manner, in comparison to inhibitors targeting individual p110 isoforms (Fig. 4*C*) (31). Finally, testing cell viability of UM cells in response to our panel of PI3K inhibitors revealed the strongest inhibition in cell viability with the pan-PI3K inhibitor, measured by cell growth over time (Fig. 4, *D* and *E*) and induction of apoptosis (Fig. 4*F*, S4*A*), indicating that UM cells are reliant on PI3K signaling for growth and survival. Ultimately, these results expand the repertoire of Gαq-FAK-regulated signaling circuitries and establish a direct connection between Gαq and PI3K/AKT *via* FAK (Fig. 4*G*).

## Discussion

The *GNAQ* oncogene is the major oncogenic driver for UM, a cancer type characterized by limited additional genetic aberrancies. As a result, UM serves as a unique model to interrogate and profile the diversity of signaling mechanisms initiated by Gαq and Gαq-coupled GPCRs to promote cell proliferation. Coupled with this, a deeper understanding of Gαq-initiated mitogenic networks provide an opportunity for the identification of novel signal transduction–based targeted therapies against UM.

Our dissection of the signaling networks regulated by Gαq led to the finding that activation of Gαq is sufficient to promote PI3K pathway. Further interrogation into the underlying mechanisms revealed that Gαq controls PI3K activation through FAK-mediated association and phosphorylation of the p85 regulatory subunit of PI3K. Finally, we demonstrate that UM cells are sensitive to genetic and pharmacological inhibition of PI3K signaling. Taken together, these findings revealed a novel signaling axis by which Gαq controls cell growth and survival by regulating the PI3K/AKT/mTOR pathway through FAK.

In this regard, Gαq has been previously linked to AKT/mTOR signaling; however, prior studies have reported varying and even paradoxical roles, suggesting that the role of Gαq in mediating PI3K/AKT signaling could be dependent on distinct cellular contexts. In exogenous overexpression systems, Gαq has been suggested to bind to and inhibit PI3K p110α, and in other settings, binding to mTOR directly and promoting the activity of mTORC1; however, the precise structural basis of these proposed mechanisms have yet to be uncovered (25, 26, 27, 28). Similarly, activity of mTOR inhibitors has been explored in *in vitro* and preclinical models of UM, but the molecular basis for these findings, as well as whether *GNAQ* activates the mTOR pathway, has not been fully investigated (32).

In general, GPCRs have been shown to signal to PI3K through the Gβγ dimers of the heterotrimeric G protein, by direct binding and activation of the p110γ/p101 heterodimer that is typically restricted to myeloid cell populations, or PI3Kβ in cells lacking p110γ (33, 34, 35, 36, 37). Our interrogation into the underlying mechanisms of Gαq oncogenic signaling network prompted us to focus our studies on endogenous contexts, enabling us to reveal key signaling components that we validated in a more generalizable HEK293-based system. In particular, focusing on UM, a cell context with persistent aberrant Gαq signaling and high FAK activity, our data support that oncogenic Gαq promotes the activation of PI3K/AKT signaling by a tyrosine phosphorylation–dependent mechanism, thereby converging with the best understood growth factor receptor tyrosine kinase signaling network. This is in alignment with our findings that Gαq activates FAK through a TRIO–RhoA–ROCK pathway (22) and prior work investigating PI3K/AKT signaling downstream RhoA (38). Future investigation regarding the specific phosphorylation sites on p85 and how these sites may integrate signals from FAK in addition to other kinases will be needed to define the precise molecular mechanisms of PI3K activation.

In this regard, our findings suggest that inhibition of all p85-associated PI3Ks may be necessary to achieve full blockade of PI3K signaling rather than individual PI3K catalytic isoforms. Indeed, this may explain why PI3Kα-specific inhibition has not been able to demonstrate significant clinical activity in UM (39). Extending this further, our findings suggest that pharmacological targeting of the pan-PI3K pathway or downstream mediators, including mTOR, may represent an attractive therapeutic strategy in UM, alone or as an approach to abrogate resistance to FAK inhibition, or as a part of multimodal targeting strategies downstream of Gαq.

Taken together, our current findings, in the context of a prior body of literature, underscore the complex and cell context–dependent molecular events underlying Gαq-driven oncogenic signaling. Indeed, other pathways identified by our screen may represent additional mechanisms that converge on FAK-mediated survival signaling driven by oncogenic Gαq. How these signaling circuitries converge with or work in parallel to the present findings have yet to be elucidated. This includes the possibility that FAK may contribute partially to ERK activation downstream from Gαq. However, the short lasting and partial effects of FAK inhibition may result from the multiple parallel pathways linking Gαq to ERK, some of which may be activated upon FAK blockade in a compensatory fashion, as suggested by our recent work (24).

The duality between canonical phospholipase C β/PKC/ERK-driven signaling and the noncanonical RhoA-dependent activation of YAP and FAK P Gαq to the direct regulation of both transient second messenger systems as well as growth-promoting transcriptional programs and tyrosine kinase–regulated phosphorylation networks (9, 22, 40). Within this framework, the activation of PI3K/AKT through Gαq may represent a novel prosurvival mechanism by which oncogenic Gαq drives cell growth and proliferation when aberrantly activated.

## Experimental procedures

### Cell lines, culture procedures, and chemicals

HEK293 cells were cultured in Dulbecco's modified Eagle's medium (D6429; Sigma–Aldrich, Inc) containing 10% fetal bovine serum (F2442; Sigma—ldrich, Inc), 1× antibiotic/antimycotic solution (A5955; Sigma–Aldrich, Inc), and 1× Plasmocin prophylactic (ant-mpp; InvivoGen). HEK293 Gαq/11 KO cells were cultured using the same conditions described previously and were a kind gift from Dr Asuka Inoue (41). UM cells (92.1, OMM1.3) were cultured in RPMI1640 (R8758; Sigma–Aldrich, Inc) containing 10% fetal bovine serum (F2442; Sigma–Aldrich, Inc), 1× antibiotic/antimycotic solution (A5955; Sigma–Aldrich, Inc), and 1× Plasmocin prophylactic (ant-mpp; InvivoGen). All cell lines were routinely tested free of mycoplasma contamination. VS-4718 (S7653), BYL719 (S2814), TGX221 (S1169), CAL101 (S2226), and BKM120 (S2247) were purchased from SelleckChem. FR900359 was prepared in the laboratory of Dr Evi Kostenis. CNO (4936) was purchased from Tocris, Inc EGF (E9644) was purchased from Sigma–Aldrich, Inc. All compounds were used at concentrations indicated in figure legends.

### Plasmids and transfections

Plasmids pCEFL-HA, pCEFL-HA-GαqQL, and pCEFL-HA-Gαq-DREADD were described previously (8). pEGFP-C1-FAK plasmid was a kind gift from Dr David Schlaepfer (42). For overexpression experiments, HEK293 cells were transfected with Turbofect (R0531; Thermo Fisher Scientific) according to the manufacturer's instructions. All knockdown experiments were performed using siRNAs purchased from Horizon Discovery Biosciences (nontargeting control: D-001810-10-05, PTEN: L-003023-00-0005, TSC2: L-003029-00-0005, GNAQ: L-008562-00-0005, FAK: L-003164-00-0005, PIK3CA: L-003018-00-0005, PIK3CB: L-003019-00-0005, PIK3CD: L-006775-00-0005), and Lipofectamine RNAiMAX Reagent (13778150; Thermo Fisher Scientific) according to the manufacturer’s instructions.

### CRISPR screen and analysis

Genome-wide CRISPR-KO screen was performed using the methods described (24). Briefly, LentiCas9-Blast plasmid was a gift from Feng Zhang (Addgene plasmid #52962) and was used to generate Cas9-expressing 92.1 UM cell line (92.1^Cas9^). The human Brunello whole-genome CRISPR pooled library was a gift from David Root and John Doench (Addgene #73178). The library contains 76,441 sgRNAs targeting 19,114 genes (four sgRNAs per gene) and 1000 nontargeting sgRNAs as the negative control.

The screen was performed by seeding 92.1^Cas9^ cells into 2245 mm × 245 mm tissue culture dishes plates (12 × 10^6^ cells/plate) divided into two treatment arms: three replicate plates for either vehicle/dimethyl sulfoxide or VS-4718 treatments. A total of 24 × 10^6^ cells were passaged into new plates containing dimethyl sulfoxide or 0.1 μM VS-4718 until the population doubling level reached 19. A total of 24 × 10^6^ cells were aliquoted from each plate at the end of the screen and stored at 80 °C for genomic DNA extraction and subsequent sgRNA quantification. The entirety of isolated genomic DNA was used for subsequent PCR to ensure capturing the full representation of the libraries. PCR products were sequenced on a HiSeq4000 instrument (Illumina) (350 million reads).

Next-generation sequencing read counts were processed and aligned using PinAPL-Py (version 2.9.2) (43). Read counts were analyzed using Mageck-MLE (0.5.9.5) (44, 45) to identify enrichment or depletion of sgRNAs in treatment *versus* control samples. Over-representation analysis of top resistance driving hits against Kyoto Encyclopedia of Genes and Genomes (46) and Biocarta (47) pathways was performed by computing statistical overlap (hypergeometric test) of all sgRNAs with positive beta score and false discovery rate <0.015 using MSigDB (version 7.5.1) (48, 49). *P* Value is derived from hypergeometric distribution, and false discovery rate *q* value was corrected for multiple hypothesis testing according to Benjamini–Hochberg method.

### Cell viability assay

Cells were seeded at a density of 8000 cells/well in 96-well white plates. Eight dilutions of each inhibitor were assayed in technical triplicates for 72 h. Cell viability was measured with the AquaBluer Cell Viability Reagent on a Spark microplate reader (Tecan). Using the GraphPad Prism, version 8.2.0 software (GraphPad Software, Inc), the half-maximal inhibitor concentration values (GI_50_) were determined from the curve using the nonlinear log (inhibitor) *versus* response–variable slope (three parameters) equation. GI_50_ values were only determined for compounds that inhibited growth by more than 50%.

### Immunoblotting and IPs

Cells were serum starved overnight and then treated according to the conditions in the figure legend. For cell lysis, cells were washed 2× in cold PBS and lysed in 1× Cell Lysis buffer (Cell Signaling Technologies; catalog no.: 9803) supplemented with Halt Protease and Phosphatase Inhibitor Cocktail (catalog no.: 78440; Thermo Fisher Scientific) and 1 mM sodium orthovanadate (catalog no.: P0758S; New England Biolabs). Lysates were centrifuged at maximum speed at 4 °C, concentrations were measured using DC Protein Assay (BioRad Laboratories; catalog no.: 5000111), and lysates were prepared with addition of 4× Laemmli Sample Buffer (catalog no.: 1610747; Bio-Rad Laboratories) and boiled for 5 min at 98 °C.

For IPs, cells were lysed with IP lysis buffer (10 mM Tris–Cl [pH 8.0], 150 mM NaCl, 1 mM EDTA, 0.3% CHAPS, 50 mM NaF, 1.5 mM Na_3_VO_4_, protease/phosphatase inhibitor [Thermo Scientific], 1 mM DTT, and 1 mM PMSF) and centrifuged at 16,000*g* for 5 min at 4 °C. Supernatants were incubated with primary antibody overnight at 4 °C, and protein A conjugated Sepharose beads for 1 h at 4 °C. Beads were washed three times with lysis buffer and prepared with addition of 4× Laemmli Sample Buffer (catalog no.: 1610747; Bio-Rad Laboratories) and boiled for 5 min at 98 °C.

For immunoblotting, cell lysates were subjected to SDS–PAGE on 10% acrylamide gels and electroblotted to polyvinylidene difluoride membranes. Blocking and primary and secondary antibody incubations of immunoblots were performed in Tris-buffered saline + 0.1% Tween-20 supplemented with 5% (w/v) bovine serum albumin or 5% w/v skim milk. Primary antibodies were all purchased from Cell Signaling Technologies and used at 1:1000 dilution. FAK (catalog no.: 71433), pY397-FAK (catalog no.: 8556), PTEN (catalog no.: 9188), TSC2 (catalog no.: 4308), AKT (catalog no.: 4691), pS473-AKT (catalog no.: 4060), S6 (catalog no.: 2317), pS235/236 S6 (catalog no.: 4858), ERK1/2 (catalog no.: 9102), pT202/Y204-ERK1/2 (catalog no.: 4370), GAPDH (catalog no.: 5174), beta-actin (catalog no.: 4970), vinculin (catalog no.: 13901), p-Tyrosine (catalog no.: 8954), p85 (catalog no.: 4257), p110α (catalog no.: 4249), p110β (catalog no.: 3011), and p110δ (catalog no.: 34050). Horseradish peroxidase–conjugated goat anti-rabbit and antimouse immunoglobulin G (Southern Biotech) were used at a dilution of 1:30,000, and immunoreactive bands were detected using Immobilon Western Chemiluminescent horseradish peroxidase substrate (Millipore) according to the manufacturer’s instructions.

### CaspaseGlo3/7 assay

Cells were seeded at a density of 10,000 cells/well in 96-well white plates. After 24 h, drug treatment or vehicle was added, and cells were assayed as indicated. Apoptosis was measured using the Promega CaspaseGlo3/7 Assay System (G8090) as per the manufacturer’s instructions.

### Statistical analysis

All data analyses were performed using GraphPad Prism, version 9.4.0 for Mac. The data were analyzed by one-way ANOVA test with correction for multiple comparison or *t* test (*asterisks* denote: ∗*p* < 0.05, ∗∗*p* < 0.01, ∗∗∗*p*< 0.001, and ∗∗∗∗*p*< 0.0001). All experiments were repeated independently with similar results at least three times.

## Data availability

All data associated with this study are presented within the article. CRISPR screen sequencing files have been deposited to the National Center for Biotechnology Information Sequence Read Archive under the BioProject accession number: PRJNA902794. Further information and requests for resources and reagents should be directed to and will be fulfilled by the lead contact, Dr J. Silvio Gutkind (sgutkind@health.ucsd.edu).

## Supporting information

This article contains supporting information.

## Conflict of interest

O.H. is an employee of Zentalis Pharmaceuticals, D.N. is an employee of TwinStrand Biosciences, unrelated to this study. P.M. is a scientific cofounder of Shape Therapeutics, Boundless Biosciences, Navega Therapeutics, and Engine Biosciences. The terms of these arrangements have been reviewed and approved by the University of California, San Diego in accordance with its conflict of interest policies. J.S.G. is consultant for Domain Therapeutics, Pangea Therapeutics, and io9, and founder of Kadima Pharmaceuticals, outside the submitted work. All other authors declare that they have no conflicts of interest with the contents of this article.
