# Supplementary material for: Whole-genome CRISPR screening identifies PI3K/AKT as a downstream component of the oncogenic GNAQ–focal adhesion kinase signaling circuitry
Source: J Biol Chem. 2022 Dec 31;299(2):102866. doi: 10.1016/j.jbc.2022.102866 (PMC9922814; doi:10.1016/j.jbc.2022.102866)

**Figure S1**

**A**

| Gene Set Name | p-value | FDR |
|---|---|---|
| Biocarta MTOR Pathway | 4.22 $e^{-6}$ | 2.02 $e^{-3}$ |
| KEGG Ubiquitin Mediated Proteolysis | 6.57 $e^{-5}$ | 1.57 $e^{-2}$ |
| KEGG WNT Signaling Pathway | 1.22 $e^{-4}$ | 1.94 $e^{-2}$ |
| Biocarta VDR Pathway | 2.34 $e^{-4}$ | 2.8 $e^{-2}$ |
| KEGG Tight Junction | 5.64 $e^{-4}$ | 4.72 $e^{-2}$ |
| KEGG Phosphatidylinositol Signaling System | 5.99 $e^{-4}$ | 4.72 $e^{-2}$ |
| Biocarta MAPK Pathway | 7.62 $e^{-4}$ | 4.72 $e^{-2}$ |
| Biocarta P38 MAPK Pathway | 7.91 $e^{-4}$ | 4.72 $e^{-2}$ |
| KEGG TGFβ Signaling Pathway | 9.54 $e^{-4}$ | 4.97 $e^{-2}$ |
| KEGG Ribosome | 1.04 $e^{-3}$ | 4.97 $e^{-2}$ |

**B**

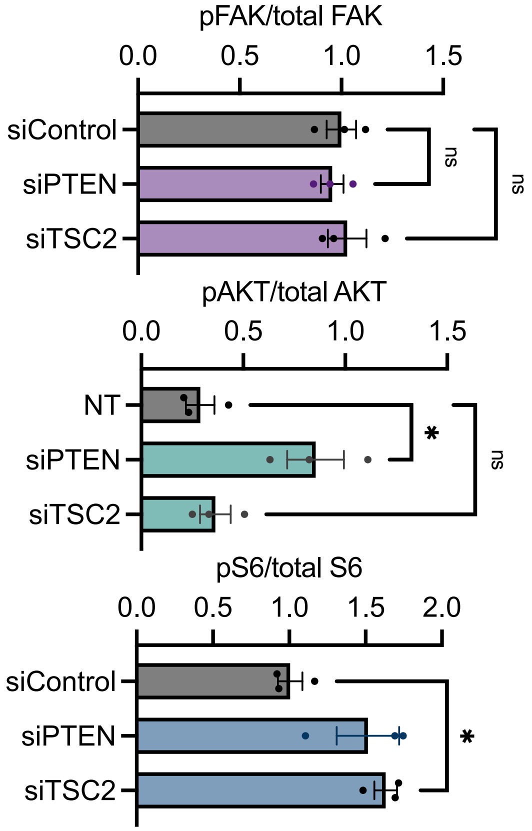

Supplement: Supporting Figure 1 [file mmc1.pdf]
