# Supplementary figures and images for: Whole-genome CRISPR screening identifies PI3K/AKT as a downstream component of the oncogenic GNAQ–focal adhesion kinase signaling circuitry
Source: J Biol Chem. 2022 Dec 31;299(2):102866. doi: 10.1016/j.jbc.2022.102866 (PMC9922814; doi:10.1016/j.jbc.2022.102866)

**Figure S2**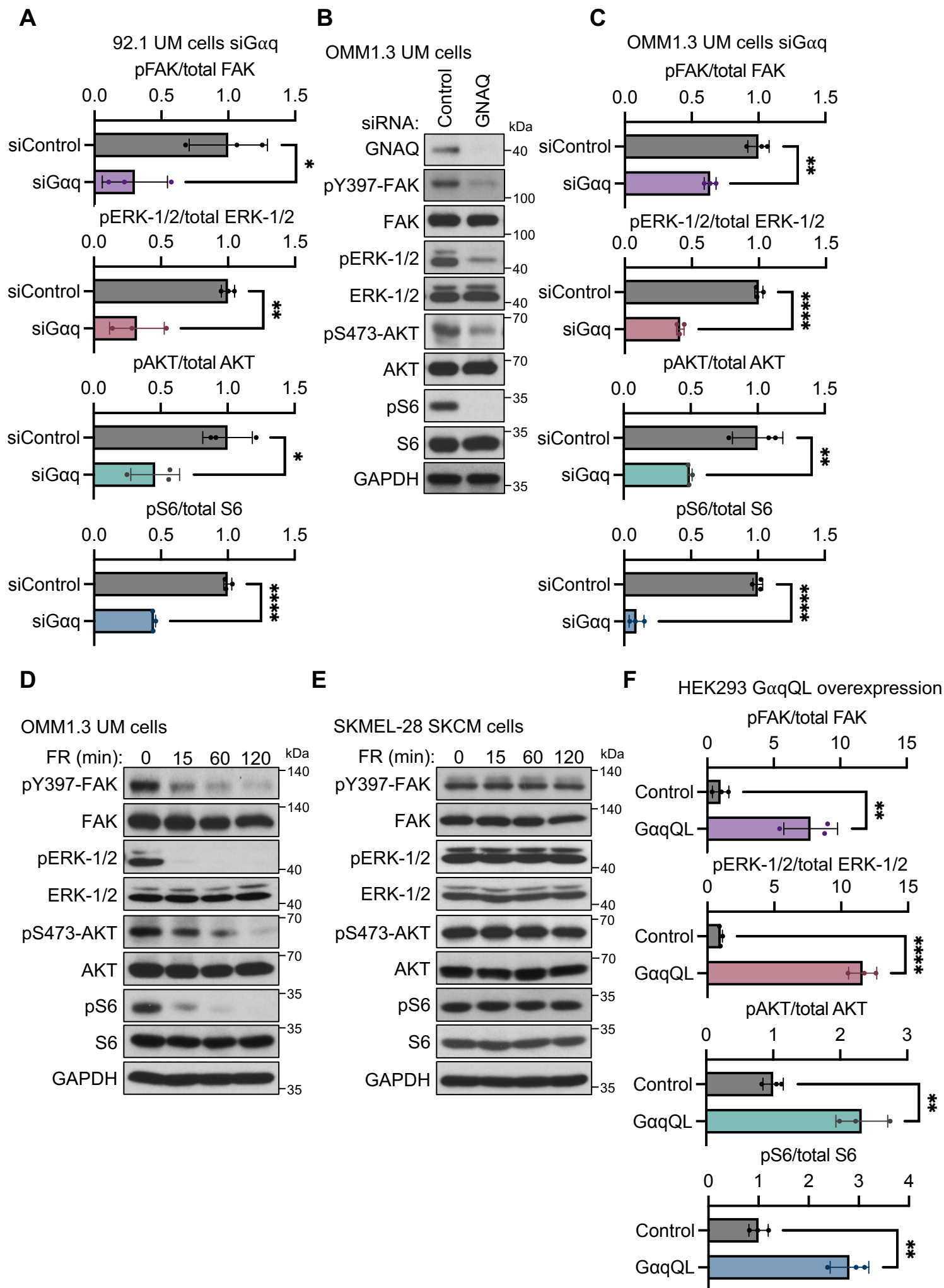

Supplement: Supporting Figure 2 [file mmc2.pdf]

**Figure S3****A**

HEK293 FAK overexpression

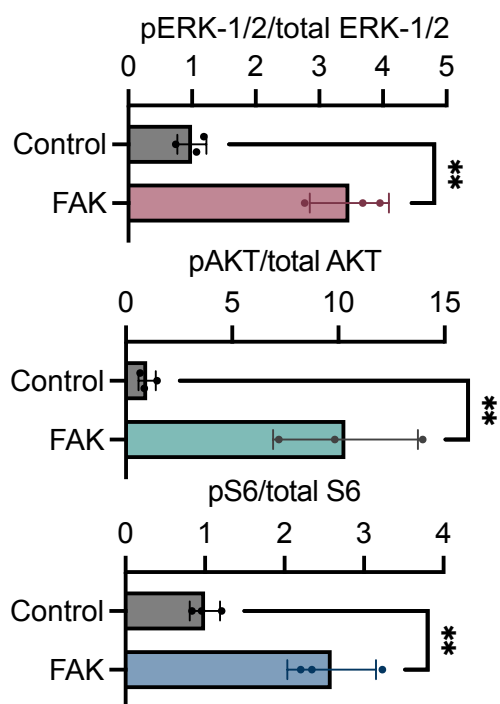**B**

92.1 UM cells siFAK

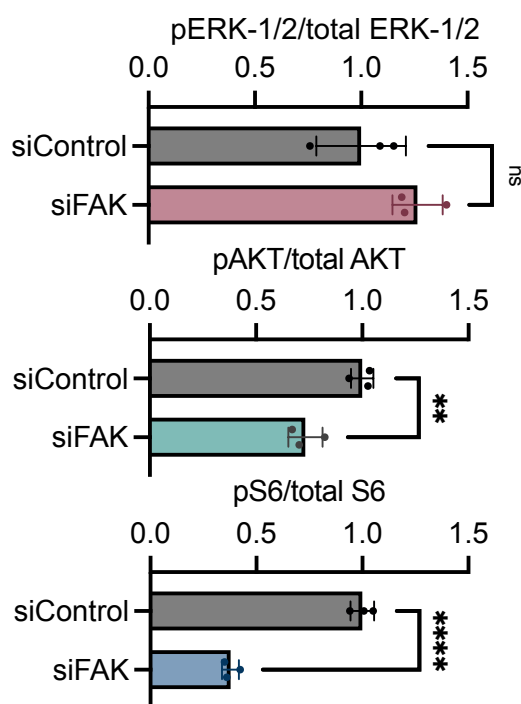**C**

OMM1.3 UM cells siFAK

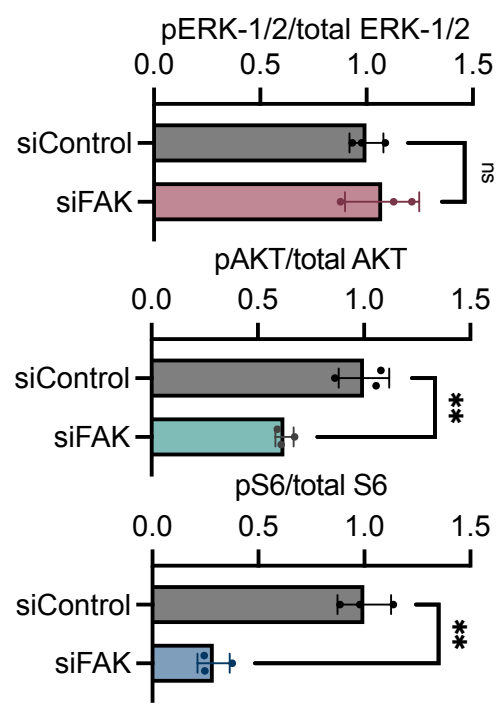**D**

OMM1.3 UM cells

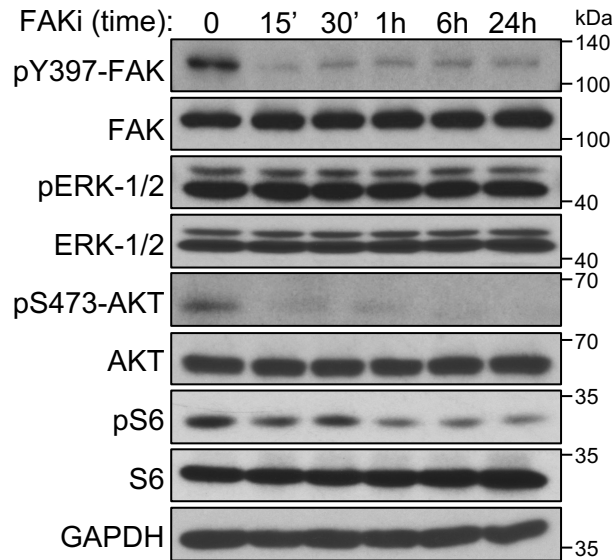

Supplement: Supporting Figure 3 [file mmc3.pdf]

Figure S4

A

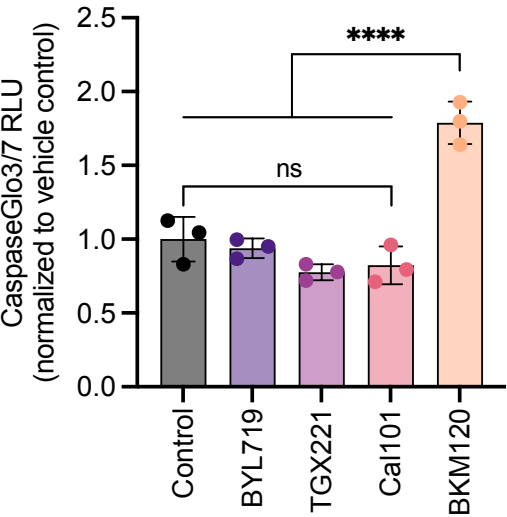

Supplement: Supporting Figure 4 [file mmc4.pdf]
